# Overexpression of miR-203 increases the sensitivity of NSCLC A549/H460 cell lines to cisplatin by targeting Dickkopf-1

**DOI:** 10.3892/or.2022.8461

**Published:** 2022-12-12

**Authors:** Ruirui Cheng, Chunya Lu, Guojun Zhang, Guowei Zhang, Guoqiang Zhao

Oncol Rep 37: 2129–2136, 2017; 10.3892/or.2017.5505

Following the publication of this paper, it was drawn to the Editors' attention by a concerned reader that certain of the mouse images shown in Fig. 5A and the flow cytometric assay data shown in Fig. 4A and B were strikingly similar to data appearing in different form in other articles written by some of the same authors, but which had already been published elsewhere or were already under consideration for publication, prior to this paper's submission to *Oncology Reports.* In view of the fact that these apparent duplications of data have come to light, the Editor of *Oncology Reports* has decided that this paper should be retracted from the Journal. The authors were asked for an explanation to account for these concerns, but the Editorial Office did not receive a satisfactory reply. The Editor apologizes to the readership for any inconvenience caused.

